# Budding uninhibited by benzimidazoles-1 (BUB1) regulates EGFR signaling by reducing EGFR internalization

**DOI:** 10.18632/aging.204820

**Published:** 2023-07-03

**Authors:** Shyam Nyati, Grant Young, Corey Speers, Mukesh K. Nyati, Alnawaz Rehemtulla

**Affiliations:** 1 Department of Radiation Oncology, University of Michigan, Ann Arbor, MI 48109, USA; 2 Department of Radiation Oncology, Henry Ford Health System, Detroit, MI 48202, USA; 3 Department of Radiation Oncology, UH Seidman Cancer Center, University Hospitals Case Medical Center, Case Western Reserve University, Cleveland, OH 44106, USA

**Keywords:** BUB1, EGFR, cancer, signaling, endocytosis

## Abstract

EGFR signaling initiates upon ligand binding which leads to activation and internalization of the receptor-ligand complex. Here, we evaluated if BUB1 impacted EGFR signaling by regulating EGFR receptor internalization and activation. BUB1 was ablated genomically (siRNA) or biochemically (2OH-BNPP1) in cells. EGF ligand was used to initiate EGFR signaling while disuccinimidyl suberate (DSS) was used for cross linking cellular proteins. EGFR signaling was measured by western immunoblotting and receptor internalization was evaluated by fluorescent microscopy (pEGFR (pY1068) colocalization with early endosome marker EEA1). siRNA mediated BUB1 depletion led to an overall increase in total EGFR levels and more phospho-EGFR (Y845, Y1092, and Y1173) dimers while the amount of total EGFR (non-phospho) dimers remained unchanged. BUB1 inhibitor (BUB1i) decreased EGF mediated EGFR signaling including pEGFR Y845, pAKT S473 and pERK1/2 in a time dependent manner. Additionally, BUB1i also reduced EGF mediated pEGFR (Y845) dimers (asymmetric dimers) without affecting total EGFR dimers (symmetric dimers) indicating that dimerization of inactive EGFR is not affected by BUB1. Furthermore, BUB1i blocked EGF mediated EGFR degradation (increase in EGFR half-life) without impacting half-lives of HER2 or c-MET. BUB1i also reduced co-localization of pEGFR with EEA1 positive endosomes suggesting that BUB1 might modulate EGFR endocytosis. Our data provide evidence that BUB1 protein and its kinase activity may regulate EGFR activation, endocytosis, degradation, and downstream signaling without affecting other members of the receptor tyrosine kinase family.

## INTRODUCTION

The epidermal growth factor receptor (EGFR) is the prototypal member of the receptor tyrosine kinase (RTK) family. EGFR is expressed in many cell types, including those of epithelial or mesenchymal origins. The RTK family members initiate multiple signal transduction cascades that modulates cell proliferation, survival, migration, invasion, differentiation, transformation and angiogenesis [1]. Due to its important role in cell proliferation and other cellular processes, EGFR remains an attractive target for cancer therapy including for radio- and chemo- radiosensitization [2, 3].

The EGFR signaling cascade is initiated by the binding with a ligand (e.g. EGF) to the receptor (EGFR) which leads to autophosphorylation, dimerization, activation of the signal mediator proteins, internalization, and eventual recycling or degradation [4]. The spatial coordination between two receptor molecules has been implicated in the activation of EGFR dimers [5]. It is suggested that binding of one kinase molecule of EGFR to a second kinase molecule asymmetrically leads to stimulation of kinase activity and enhanced autophosphorylation which is a crucial factor for receptor endocytosis. Disruption of asymmetric dimer interface of EGFR leads to reduction in its kinase activity and autophosphorylation in ligand-stimulated cells.

EGFR has more than 20 Tyr residues that can be phosphorylated [6]. Ligand binding can induce homo- or hetero-dimerization, which can activate many sites within the C-terminus such as Y992, Y1045, Y1068, Y1086, Y1148 and Y1173, or SRC non-receptor kinase can phosphorylate Y845 and Y1101. EGFR phosphorylation at Y845 in the kinase domain is implicated in stabilizing the activation loop, maintaining the active state enzyme, and providing a binding surface for substrate proteins. Phosphorylation at 1173 creates a major binding site for the protein tyrosine phosphatase SHP-1, which can dephosphorylate EGFR and thereby block EGFR-induced activation of the ERK1/2 signaling pathway. Autophosphorylation at Y1092 and/or Y1110 recruits STAT3 (see review [3] for details).

Two different models have been proposed for the activation of EGFR by ligand binding. According to the “ligand-induced dimerization model”, EGFR exists as monomers and gets dimerized upon ligand binding. This brings intracellular kinase domains into vicinity for trans-autophosphorylation to activate downstream signaling. According to the “rotation model”, EGFR exists as an inactive dimer and ligand binding induces rotation of the transmembrane domains, which reorients the symmetric inactive kinase domain dimer to an asymmetric active form. Advancement in structural studies have yielded insight that supports the “rotation model” that EGFR exists as inactive dimers prior to ligand binding [7–10].

The EGFR inhibition with tyrosine kinase inhibitors and monoclonal antibodies has shown improvement in outcome in subsets of patients with head and neck, lung, and colorectal carcinomas [11, 12]. We and others have shown that EGFR stability plays a key role in cell survival after chemotherapy and radiotherapy [13]. We also showed that HSP90 [14] and SMURF2 [15] maintain stability of wild-type EGFR in cancer cells and tumors. Moreover, by designing a short peptide that blocks the EGFR binding to HSP90 we showed that this peptide disrupted not only the EGFR-HSP90 interaction but also EGFR dimerization [16]. Recently, Sortilin [17] and Mitogen-inducible gene 6 (MIG6) and Sprouty 2 (SPRY2) were identified to interact with EGFR and regulate EGFR trafficking [18]. The mechanisms that direct the internalization and compartmentalization of activated EGFR for signaling turnover or signaling activation are largely known [19]. EGFR is known to signal not only at the cell surface, where ligand engagement occurs, but it continues to signal during endocytosis for considerable period depending on the context [20, 21].

BUB1 (budding uninhibited by benzimidazoles-1) is a Ser/Thr kinase which plays a key role in mitotic spindle checkpoint assembly and chromosome congression [22, 23]. We identified BUB1 as an essential mediator of TGFβ signaling [24–27] through genomic screen. Recently, we and others have reported novel non-cell-cycle related functions of BUB1, including TGFβ signaling [27], telomere DNA replication [28], DNA damage response [29, 30], and viral entry [31]. Recent studies using two hybrid screens [32] as well as siRNA screens [33] have identified novel roles of BUB1 beyond chromatid segregation and provided proof that BUB1 serve as a component of signaling micro-domains within membranes. Several studies identified that BUB1 directly interact with key proteins required for endocytosis including supervillin [32], Vps5 and β2-adaptins [34, 35]. Other studies discovered a crucial role for BUB1 in viral infection through clathrin-dependent endocytosis of Drosophila C virus (DCV) and vesicular stomatitis virus (VSV) [31]. BUBR1 which is a pseudokinase and a BUB1 paralog has also been shown to interact with β-adaptin (AP2B1) and play a critical role in insulin receptor endocytosis [36]. Very recently Bub1-Bub3 complex has been shown to regulate autophagosome-mediated macrolipophagy in Drosophila [37].

These studies provided a strong rationale for our hypothesis that BUB1 regulates EGFR dimerization and thus EGFR signaling at membrane micro-domains wherein extracellular signals are communicated to the intracellular network. We postulate that BUB1 helps in the formation and stabilization of EGFR dimers at the membrane and may regulate endocytosis of activated EGFR into either clathrin dependent (EEA1 coated) or independent (caveolin coated) vesicles thus impacting receptor recycling or degradation and subsequently signaling amplitude and duration [38, 39].

## MATERIALS AND METHODS

### Antibodies and reagents

Antibodies to pAKT (cat# 4060), total AKT (cat# 9272), pEGFR(Y845) (cat #2231), pEGFR(Y1092), pEGFR (Y1068) (cat# 3777), pEGFR (Y1173), total EGFR (cat # 4267; # 2232), Her2/ErbB2, c-Met, pFAK, FAK, pErk1/2 (42/44) (cat # 9101; cat # 4370), Erk1/2 (42/44) (cat # 4695) and GAPDH (all from Cell Signaling), β-adaptin (BD Biosciences, cat # 610382), EEA1 (Ab70521), and Actin were from Abcam. Total EGFR antibody (Sc-03) was from SantaCruz Biotechnology. The HRP-conjugated secondary antibodies were from Jackson ImmunoResearch. Recombinant human EGF (AF-100-15) was obtained from PeproTech, custom BUB1 siRNA, as well as non-silencing siRNA (NSS) were obtained from GE-Dharmacon. SD208 (Tocris), Erlotinib (gift from Genentech), Cetuximab (Bristol Myer Squibb), 2OH-BNPP1 was synthesized in-house as described by Jiang et al., [40].

Cycloheximide was obtained from Acros Organics/Fischer Scientific, while DSS was from ThermoFisher Scientific. FuGENE 6 was from Roche while Lipofectamine 2000 was from Invitrogen/ThermoFisher Scientific. ProLong Gold anti-fade mounting media (without DAPI) was purchased from Invitrogen/ThermoFisher Scientific.

### Cell culture and transfection

The human lung carcinoma cell line A549 and lung epithelial cell line MRC5, normal kidney cell line HEK293T were obtained from American Type Culture collection (ATCC). NCI-H358 cell line was a gift from Dr. David Beer (U of Michigan) while breast cancer cell-line MDA-MB-231 derived line 1833 was a kind gift from Dr. Joan Massague (Memorial Sloan Kettering Institute, NY, [41, 42]. Cells were maintained in RPMI-1620 or DMEM media supplemented with 10% heat-inactivated fetal bovine serum, 1% glutamine, and 0.1% penicillin/streptomycin/gentamycin (GIBCO-Invitrogen). Cells were grown in a humidified incubator at 37° C and 5% CO2. siRNAs were transfected using Lipofectamine 2000.

### Western blot analysis

Western analysis was carried out using standard protocols. Cells were grown in culture dishes, transfected with specific siRNA, or treated with select compounds and EGF for designated time periods. Cell lysates were prepared in IP-lysis buffer (50mM Tris PH 7.4, 1% NP40, 0.25% Deoxycholate sodium salt, 150mM NaCl, 10% Glycerol, and 1mM EDTA) supplemented with 1X PhosStop (Roche), 1X Protease inhibitor cocktail (Roche), Sodium Ortho Vanadate, Sodium fluoride, PMSF, and β-Glycerol phosphate (2 μM each). Protein amount was estimated using detergent compatible Dc assay kit (BioRad) and equal amount of protein was resolved on 3-8% Tris-Acetate or 4-12%-Bis-Tris gels and transferred to PVDF membranes according to manufacturer’s recommendation. Membranes were blocked for 1 hours at room-temperature with 5% milk-TBST or 5% BSA-TBST. Membranes were incubated over-night at rotating platform with specific primary antibodies (usually 1:1000 dilutions). Membranes were washed three times with TBST, followed by incubation with HRP-conjugated secondary antibodies (1 hour at room temperature) then visualized using the Enhanced Chemiluminescence (ECL) Western Blotting System (GE Healthcare).

### EGFR dimer formation assay

For dimer formation assays, cells were pre-treated with vehicle (DMSO), 2OH-BNPP1, erlotinib (10 μM) for 30-60 minutes, followed by EGF (30-50 ng/mL) for 10 minutes. Cells were then treated with freshly made DSS (200 μM) for 30 minutes with intermittent shaking/swirling every 5 minutes. After the treatment cells were washed twice with ice-cold PBS and lysed in 1.2X Laemmli direct lysis buffer (4X Laemmli diluted with IP-lysis buffer containing protease and phosphatase inhibitors). Cell lysates were supplemented with 2-5% b-mercaptoethanol and sonicated. Lysates were boiled at 95C for 5-7 minutes and loaded on 3-8% Tris-Acetate gels. Proteins were transferred to PVDF membrane and western blotting was performed as described above.

### Protein half-life assay

For Protein half-life studies cells were serum-starved over-night. Next morning cells were treated with vehicle (DMSO) or inhibitors (10 μM) and Cycloheximide (50 μM) mixture for an hour in serum-free media. Cells were then treated with EGF (30-50 ng/mL) without removing inhibitors and Cycloheximide. Samples were harvested at different time-points following EGF treatment. Total protein lysates were made using 1.2X direct lysis buffer and run on gels. Alternatively, lysates were made in IP-Lysis buffer and equal amount of protein was run on SDS-PAGE gels for western-analysis.

### Confocal microscopy

MDA-MB-231-1833 cells (100-125k/well) were plated in 6-well plates containing 4-5 glass coverslips (1.5mm thick, 12 mm dia). Alternatively, cells were plated on glass chamber slides (15-20K/chamber). Cells were allowed to attach to the glass surface for two days. Cells were serum starved for 3 hours. Starved cells were pretreated for 60 minutes with 10 μM 2OH-BNPP1 or erlotinib or vehicle (DMSO) in pre-warmed serum free RPMI. This was followed by an additional treatment with 50 ng/mL EGF/vehicle for multiple time points ranging from 5 minutes to 80 minutes. Cells were incubated at 37° C during drug treatment to allow signaling to continue and receptors to get internalized. Coverslips were taken out and washed with ice-cold PBS and fixed with ice cold 10% buffered formaldehyde for 20 minutes (on ice). Cover glasses or slides were rinsed with cold PBS and further fixed using cold Methanol for 20 minutes in -20C freezer. Cells were rehydrated by gently rocking/washing 3 times (5 minutes each) with PBS and permeabilized on ice with PBS-T (0.5% Triton X-100) for 5 minutes. Cover glasses were rinsed 3 times with PBS and blocked for 2 hours with gentle rocking with a blocking buffer containing 2% BSA, 5% donkey serum, and 10% goat serum in PBS at room temperature. Antibodies were diluted in blocking buffer and incubated either at room temperature for 1 hour or over-night at 4° C in humidified chamber with gentle rocking. EEA1 antibody was diluted 1:2000 (mouse, Abcam) and pEGFR1068 (1:200, Rb, Cell Signaling). Cover-glasses were rinsed 3X with PBS and washed 3X 10 minutes with PBS-Tween 20 (0.025%) at room temperature. Secondary antibodies (αRb-647 1:500 or 1:1000 and αM-488 1:200) were made in blocking buffer and incubated for 1 hour at room temperature in dark. Cover slips were rinsed, washed, and mounted using ProLong Gold without DAPI, sealed with clear nail paint and allowed to dry over-night at room temperature in dark. Confocal microscopy was performed on an Olympus confocal microscope at the Microscopy and Image Analysis Laboratory (MIL) core at University of Michigan. Images were acquired using a 60X oil objective lens, usually images were acquired only for a single plane. At least one image with 2X magnification was also acquired for each treatment and time point.

### Protein thermal stability assay (PTSA)

To confirm that BUB1 inhibitor 2OH-BNPP1 did not directly bind to and inhibit EGFR, thermal stability assay (TSA) was performed. TSA can be utilized to evaluate small molecule and target engagement [43, 44]. This assay is based on the principle that binding of a small molecule to a protein can lead to thermal stabilization or even destabilization which results in the protein melting curve shifting. Varying concentrations of 2OH-BNPP1 (300 nM to 10 μM) or osimertinib (300 nM- 3 μM, positive control) were added with 100 ng purified recombinant EGFR kinase domain in PCR tubes and incubated for 30 minutes at 4° C. After incubation, tubes were incubated at 47° C for 3 minutes in thermal cycler (Eppendorf). Tubes were centrifuged, and supernatant was collected. This supernatant was mixed with protein loading dye and resolved on SDS-PAGE gels and probed with an anti-EGFR antibody.

### Data analysis

Correlation analysis for BUB1 and EGFR were performed using log2 median centered gene expression values from the TCGA lung dataset [45]. Expression data was obtained from Oncomine.org [46] and coexpression analysis done using MedCalc software with Pearson’s correlation coefficient calculated. Western blots were scanned using Epson Perfection 4490 Photo scanner and band densitometry analysis was performed on ImageJ. Statistical analysis and data plotting was done in MS Excel and GraphPad Prism. The quantitation of the co-localization of pEGFR with EEA1 was performed using an object-based plugin “Just Another Colocalization Plugin” (JACOP) on ImageJ [47]. Manders’ overlap coefficient is based on the Pearson’s correlation coefficient with average intensity values being taken out of the mathematical expression. Manders’ overlap coefficients M1 and M2 were estimated which vary from 0 (0% co-localization) to 1 (100% co-localization) between two images. M1 (fraction of image A overlapping with B) and M2 (fraction of image B overlapping with A) values were estimated with auto threshold as well as with user defined threshold. User defined threshold was used to reduce the background noise since Manders’ coefficient is very sensitive to noise. The fraction of pEGFR Y1068-red overlapping with EEA1-green (M2 values) acquired after noise subtraction is plotted. Additionally, the extend of colocalization between EEA1 and pEGFR was also estimated using MBF ImageJ (Image J for Microscopy, formerly WCIF (Wright Cell Image Facility) ImageJ) and colocalization plots for visual representations.

### Data availability

The data can be shared upon request.

## RESULTS

### Knockdown of BUB1 leads to an increase in active EGFR (pEGFR) dimers

**Figure 1:**
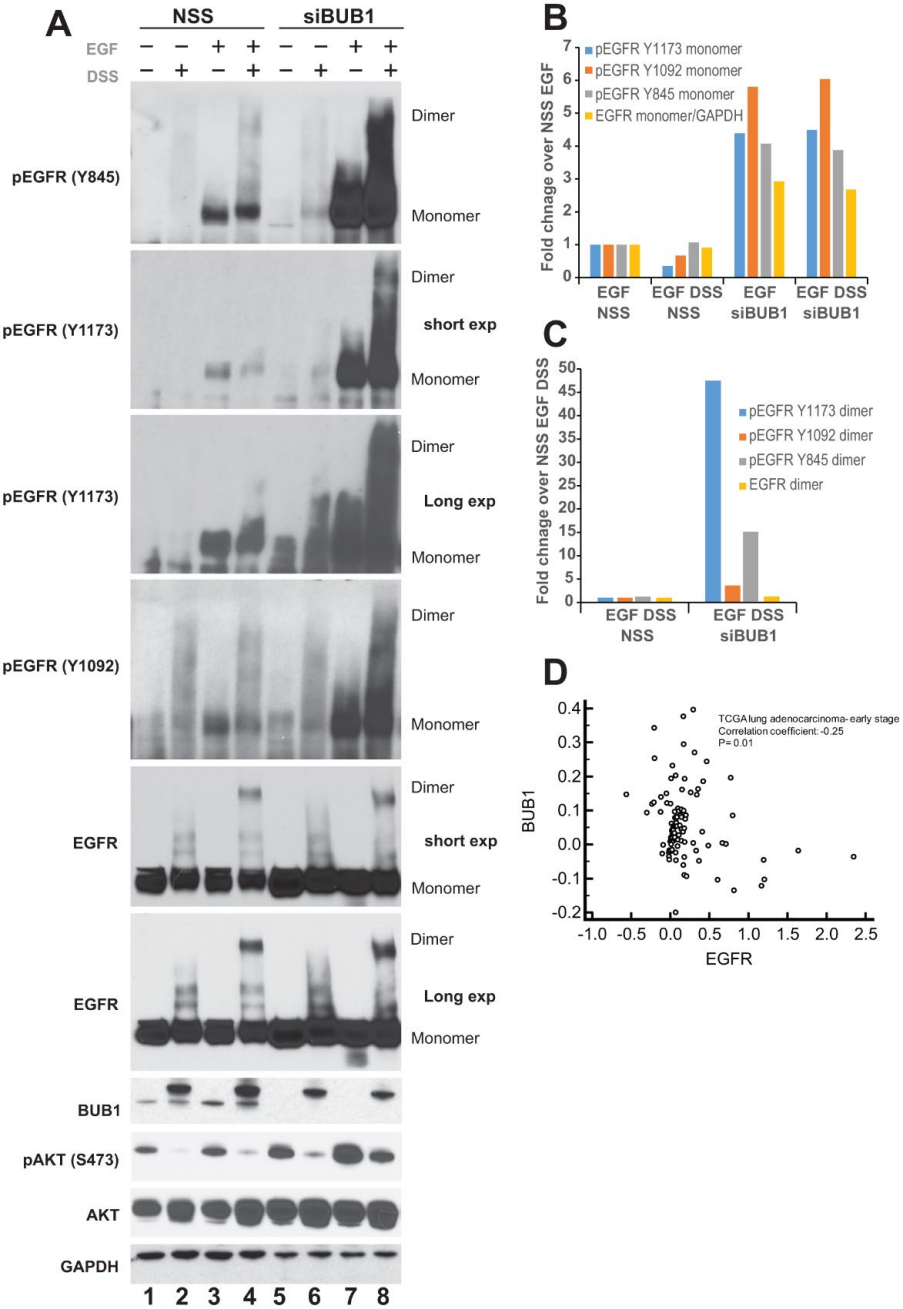
**BUB1 depletion stabilizes EGFR.** (**A**) A549 cells were transfected with non-targeting control scrambled (NSS) siRNA or BUB1siRNA. 48 hours post-transfection cells were cross-linked with 100 μM DSS for 30 minutes followed by 30 ng/mL EGF for an additional 30 minutes. Resulting lysates were resolved on SDS-PAGE gels and probed with indicated antibodies. (**B**) Quantitation of western blots from (**A**) only the monomer species of EGFR is plotted. Control siRNA (NSS) transfected, EGF treated lanes were set as 1 fold and used as a baseline for estimating fold enrichment in other samples. (**C**) Quantitation of pEGFR and EGFR dimers from SDS and EGF treated lanes only (lanes 4 and 8 only in **A**). NSS transfected lane was set as 1 fold and used as a baseline for estimating fold enrichment in BUB1 siRNA transfected samples. (**D**) Gene expression values from non-metastatic adenocarcinoma samples (N=331) from TCGA lung dataset were log_2_ transformed and median centered and correlation coefficient (r) was calculated. BUB1 and EGFR expression is expressed as log_2_ transformed values. Correlation coefficient and p-value are listed.

To demonstrate a requirement of BUB1 protein on EGFR signaling, A549 lung adenocarcinoma cells were transfected with non-targeting scrambled siRNA (NSS; control) or siRNA specific to BUB1. These cells were serum starved over-night and signaling was initiated by addition of EGF for 30 minutes (30 ng/mL). Since activation of EGFR signaling involves dimerization of two EGFR molecules [48], disuccinimidyl suberate (DSS) cross-linking agent was used to cross-link the EGFR dimers for evaluation. As expected, no dimers were observed in DSS only treated lanes (Figure 1A, lanes 2 and 6). Addition of EGF lead to dimerization of EGFR receptors (total EGFR as well as pEGFR; Figure 1A, lane 4) in NSS transfected lane. Most strikingly, we observed significant increase in EGF mediated EGFR phosphorylation in BUB1 depleted lanes (Figure 1A, lanes 7, and 8). Moreover, addition of DSS showed elevated pEGFR dimers (Figure 1A, lane 8) without significant increase in total EGFR dimers (Figure 1A, lane 8). The increase in phospho-EGFR was observed with all the antibodies tested including Y845 (Src mediated phosphorylation; active state EGFR), Y1092 (auto phosphorylation; Stat3 binding site) and Y1173 (autophosphorylation; SHP1 phosphatase binding site).

Western blot quantitation for monomers and dimers were performed using ImageJ and plotted separately. For monomer plot the data was normalized (set as 1) to control siRNA transfected EGF treated lane (lane 3 in Figure 1A). Addition of cross-linking agent DSS in NSS transfected samples showed marginal decrease in pEGFR Y1092 and Y1173 monomers (Figure 1B) while no change was observed in pEGFR Y845 monomers or total EGFR monomers (Figure 1B). Cells wherein BUB1 was depleted by using siRNA showed between 2-6 folds increase in phospho- as well as total EGFR monomers (Figure 1B). For quantitation of dimer species, the data was normalized (set as 1) to control siRNA transfected, EGF and DSS treated samples (lane 4 in Figure 1A). We observed between 4 to 40-fold increase in pEGFR dimers in BUB1 siRNA transfected cells (Figure 1C) while only about 2-fold enrichment in the total EGFR dimer species was observed (Figure 1C).

Next, we evaluated if the kinase activity of BUB1 played a role in the observed increase in EGFR levels and activation (Figure 1). A549 cells were treated with vehicle (DMSO) or 2OH-BNPP1, a small-molecule inhibitor of BUB1 at 5 μM or 10 μM for 48 hours. EGFR tyrosine kinase inhibitor erlotinib was used as a control. Surprisingly, we did not observe any increase in total EGFR when BUB1 was inhibited by 2OH-BNPP1 (Supplementary Figure 1). As expected, erlotinib did not cause any changes in EGFR levels in A549 cells [49, 50]. This data suggests that BUB1 protein may be involved in regulation of EGFR signaling, and that this regulation could be different than BUB1’s kinase activity. Finally, to determine whether there was a correlation between BUB1 expression and EGFR expression we interrogated the TCGA lung dataset [45] for co-expression analysis. Evaluation was limited to patients with non-metastatic patients with adenocarcinoma, a disease driven by perturbed EGFR-pathways signaling. This analysis demonstrated a significant negative correlation between EGFR gene expression levels and BUB1 expression, suggesting the regulatory effects noted *in vitro* may also be relevant in tumors from human patients (Figure 1D).

### BUB1 inhibition attenuates EGFR signaling

**Figure 2:**
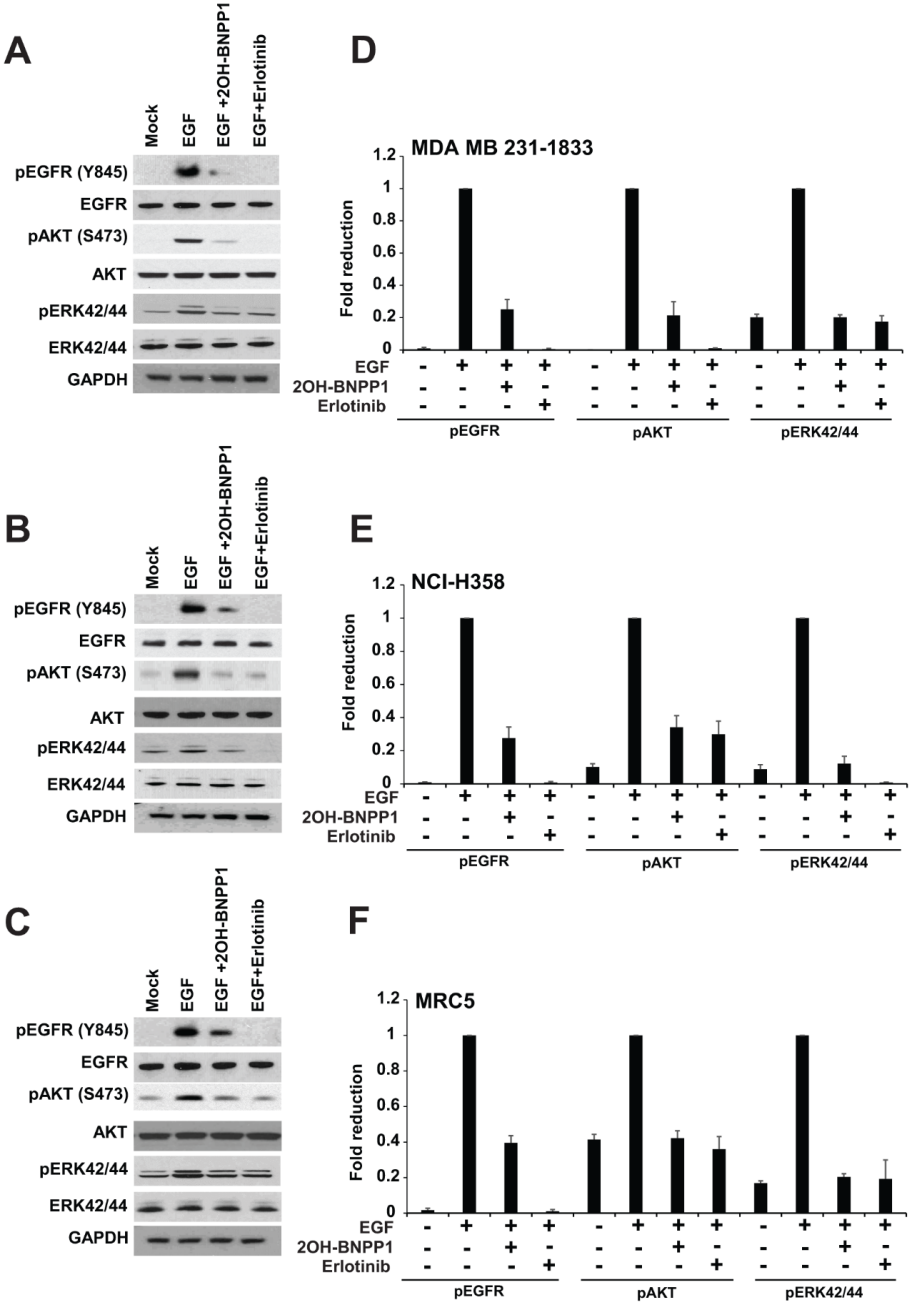
**BUB1 inhibition blocks EGFR signaling.** MDA-MB-231-1833 (**A**) NCI-H358 (**B**) and MRC5 (**C**) cells were starved and pretreated with BUB1 kinase inhibitor 2OH-BNPP1 (10 μM) or EGFR inhibitor erlotinib (10 μM) for 1 hour followed by EGF (30 ng/mL) treatment for an additional 30 minutes. Resulting lysates were run on 4-12% Bis-Tris SDS-PAGE gels, transferred to PVDF membranes and probed with indicated antibodies. (**D**–**F**) western blots for phosphorylated proteins from (**A**–**C**) were quantitated (three separate biological repeats) using ImageJ and plotted.

To evaluate the effect of BUB1 kinase activity on EGF ligand mediated EGFR signaling activation, various cell lines including MDA-MB-231-1833 (Figure 2A), NCI-H358 (Figure 2B), MRC5 (Figure 2C) were used. MDA-MB-231 is a triple negative breast cancer (TNBC) cell line which expresses EGFR and its bone-specific clone (MDA-MB-231-UR) is highly enriched for EGFR [51]. MDA-MB-231-1833 cells were repeatedly injected in mice to collect bone metastatic, very aggressive clones [41, 42]. These cells were serum starved over-night then pretreated with BUB1 inhibitor 2OH-BNPP1 or EGFR inhibitor erlotinib for 30 minutes followed by 30 minutes treatment with EGF. Resulting lysates were resolved on SDS-PAGE and probed with pEGFR (Y845) antibodies (Figure 2). As expected, EGF caused activation of EGFR and it was completely blocked by pre-treatment with erlotinib. Not surprisingly, BUB1 inhibition also showed significant reduction in EGFR activation (Figure 2A–2C). We also observed a marked reduction in downstream signaling including AKT phosphorylation (pAKT S473) and Erk42/44 phosphorylation in these cells. Quantitation of western blots from at least three independent experiments showed these changes to be consistent. To determine if the above effects of BUB1 inhibition on EGF signaling were specific, we treated MDA-MB-231-1833 cells with a TGFBR1 inhibitor SD208 and stimulated these cells with EGF (Supplementary Figure 2A). There was no effect of SD208 on EGF stimulated pEGFR Y845, pAKT S473, pErk42/44 (Supplementary Figure 2A).

Next, we evaluated if BUB1 inhibition dose-dependently decreased EGF mediated EGFR activation. A549 cells were serum starved then either treated with vehicle (mock) or treated with an increasing concentration of 2OH-BNPP1 (1 μM to 50 μM) followed by EGF treatment. Cell lysates were made 30 minutes post EGF treatment and resolved on SDS PAGE and probed with pEGFR (Y845) as well as pAKT (S473) antibodies which showed a dose-dependent decrease in EGFR and AKT activation (Supplementary Figure 2B).

### BUB1 inhibitor blocks early EGFR signaling

**Figure 3:**
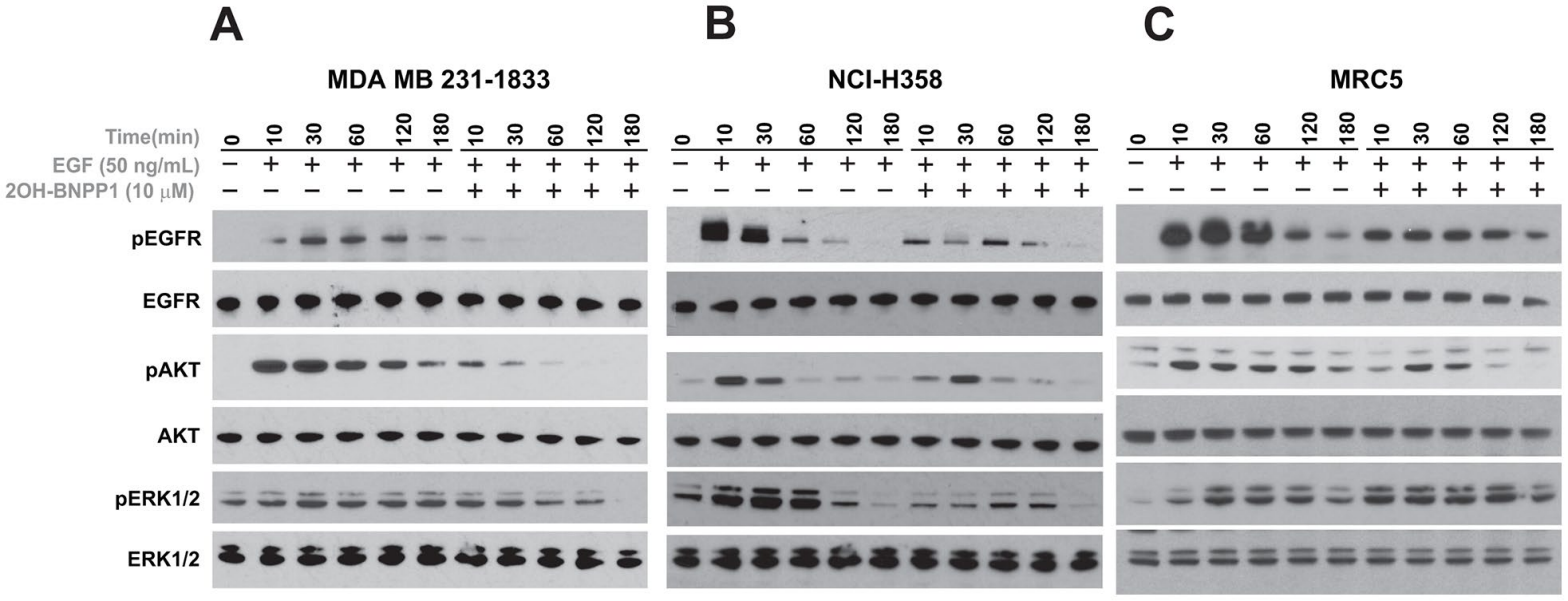
**BUB1 inhibition reduces EGFR activation.** MDA-MB-231 (**A**) NCI-H358 (**B**) and MRC5 (**C**) cells were serum starved and pre-treated with 10 μM 2OH-BNPP1 for 1 hour followed by 50 ng/mL EGF. Cells were harvested at the indicated time-points (10-180 minutes) after EGF treatment. Whole cell lysates from these samples were resolved on SDS-PAGE gels and transferred to PVDF membranes. The membranes were blocked with 5% de-fatted milk-TBST and probed with pEGFR (Y845), pAKT (S473), and pERK1/2 antibodies. The blots were also probed with antibodies raised against total proteins.

After confirming that BUB1 inhibitor 2OH-BNPP1 significantly reduces EGF mediated EGFR signaling (Figure 2), we next evaluated if inhibition of BUB1 kinase activity also affects EGFR signaling at early time points. MDA-MB-231-1833, NCI-H358 and MRC5 cells were plated and serum starved over-night. These cells were treated with EGF (50 ng/mL) or with mix of 2OH-BNPP1 (10 μM) and EGF (50 ng/mL) and harvested at various time points starting at 10 minutes post treatment. Resulting lysates were run on gels and probed with pEGFR (Y845), pAKT (S473) and pERK42/44 as well as for total proteins (Figure 3). We observed EGFR phosphorylation at the earliest time point tested (10 minutes) in EGF treated lanes which decreased over time (Figure 3). However, cells co-treated with BUB1 inhibitor showed significantly lower EGFR activation (Figure 3) which was further reduced over time in MDA-MB-231-1833 and NCI-H358 cells (Figure 3A, 3B) or was maintained at the lower activation level throughout in normal lung fibroblasts (MRC5 cells, Figure 3C). AKT activation was generally lower which decreased over time in all the cell-lines while ERK42/44 activation decreased in MDA-MB-231-1833 and NCI-H358 (Figure 3A, 3B) and maintained in MRC5 cells (Figure 3C).

### BUB1 kinase activity does not affect inactive EGFR dimerization

Based on above findings that knockdown of BUB1 though causes moderate increase in total EGFR monomers and dimers (inactive EGFR), it causes a significant increase in phospho-EGFR monomers and dimers (active EGFR) and that 2OH-BNPP1 dampens EGF mediated signaling, we sought to evaluate if BUB1 kinase activity regulated dimerization of active or inactive EGFR molecules.

**Figure 4:**
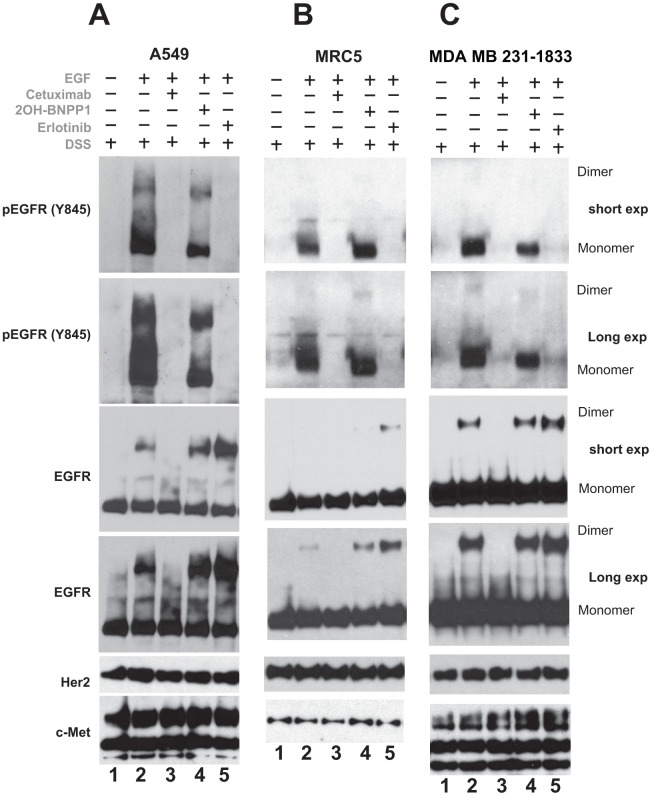
**Inhibition of BUB1 kinase activity reduces EGFR active dimers without affecting inactive-EGFR dimers.** A549 (**A**) MRC5 (**B**) and MDA-MB-231-1833 (**C**) cells were serum starved for 3-4 hours, pretreated with 2OH-BNPP1 (10 μM), erlotinib (10 μM) or cetuximab (50 μg/mL) for 1 hour followed by EGF (50 ng/mL) for 10 min. DSS (200 μM) was added for an additional 20 minutes. Total cell lysates were made, resolved on 3-8% gels and probed with pEGFR (Y845), EGFR, Her2 and c-Met antibodies.

A549, MRC5 and MDA-MB-231-1833 cells were plated, serum starved and treated with cetuximab (EGFR inhibiting antibody, which blocks dimerization of inactive as well as of active/phosphorylated-EGFR), erlotinib (blocks dimerization of phospho-EGFR only, may stabilize inactive -EGFR) and 2OH-BNPP1 for 60 minutes. Cells were then treated with EGF and DSS cross linking agent for 20 minutes. The resulting lysates were run and probed with pEGFR (Y845) as well as total EGFR antibodies. As expected, EGF caused dimerization of total and phospho-EGFR (lane1, Figure 4A–4C) which was completely blocked by cetuximab (lane 2, Figure 4A–4C). 2OH-BNPP1 marginally blocked phospho-EGFR dimers (Figure 4A) while had no effect on total EGFR dimers (Figure 4A–4C). Erlotinib on the other hand blocked active EGFR dimers completely but slightly stabilized total EGFR dimers (Figure 4A–4C). Additionally, probing of these blots with another member of EGFR family (Her2/ErbB2) and a tyrosine kinase c-Met did not reveal any changes (Figure 4A–4C), suggesting that the effect of BUB1 is specific to EGFR.

### BUB1 inhibition by 2OH-BNPP1 stabilizes EGFR

**Figure 5:**
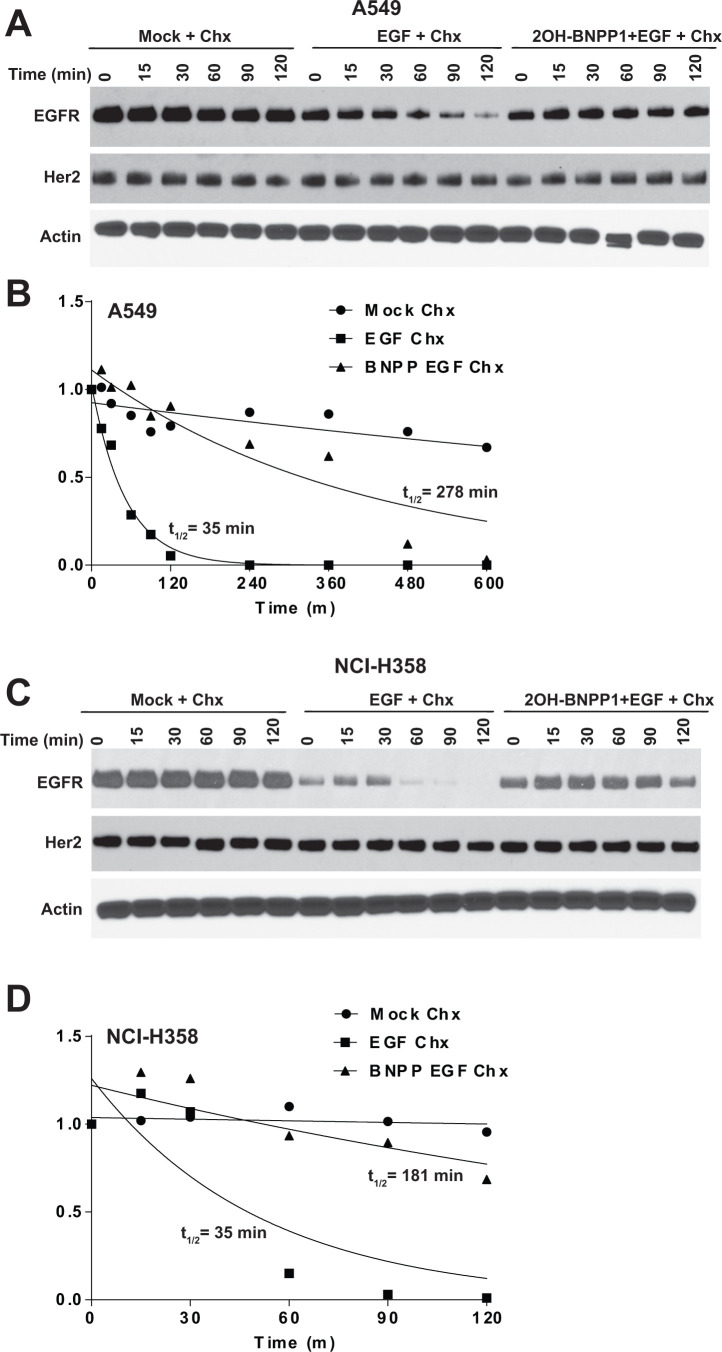
**Inhibition of BUB1 kinase activity by 2OH-BNPP1 prolongs EGFR half-life.** (**A**) A549 lung adenocarcinoma cells were treated with 2OH-BNPP1 (10 μM) and Cycloheximide (50 μg/ML) for 1 hour followed by EGF (50 ng/mL). Cells were harvested at different time points after EGF treatment and resulting lysates were run on SDS-PAGE gels and probed with EGFR, Her2 and Actin antibodies. (**B**) Densitometric analysis of EGFR blots in A549 was performed using ImageJ. Resulting data was analyzed in MS-Excel. The protein half-life plots (t1/2) were generated using GraphPad Prism. The plots are of combined data from 2-3 biological repeats is shown. (**C**, **D**) NCI-H358 cell lines was treated similar to A549 and EGFR protein half-life was estimated by densitometric analysis.

Based on our above observation that BUB1 knockdown leads to an increase in total EGFR levels, and that BUB1 inhibition reduces EGFR signaling and dimerization of active EGFR dimers, we hypothesized this should cause a reduction in internalization of EGFR thus reduced EGFR degradation. A549 and NCI-H358 cells were treated with vehicle or 2OH-BNPP1 along with cycloheximide to block the nascent protein synthesis for an hour. Cells were then treated with EGF and harvested at different time points (0, 15, 30, 60, 90 and 120 minutes). Resulting lysates were run on SDS-PAGE gels and probed with EGFR antibodies. As expected, we did not observe any changes in total EGFR levels in mock treated cells for up to 2 hours (Figure 5A, 5C) while a rapid decrease in EGFR was observed within 60 minutes post EGF treatment in A549 and NCI-H358 cells (Figure 5A, 5C). EGF mediated EGFR degradation was completely blocked by BUB1 inhibitor 2OH-BNPP1 in these cells (Figure 5A, 5C). The half-life or turnover rate (t_1/2_) of EGFR depends on the level of activation due either to presence of ligand or presence of activating mutation within EGFR [52]. In WT-EGFR cells, in the absence of EGF, the t_1/2_ of EGFR is ~18-20h [53], therefore we conducted cycloheximide chase experiments with longer time points to accurately quantitate half-life of EGFR in the presence of a BUB1 inhibitor (Supplementary Figure 3). Quantitation of EGFR from the immunoblots showed that t_1/2_ increased from 35 minutes in EGF only treatment to ~5 hours in 2OH-BNPP1+EGF treatment in A549 cells (Figure 5B). t_1/2_ for mock treatment in these cells was estimated to be ~22 hours. In NCI-H358 EGF only treated cells the t_1/2_ for EGFR was estimated to be 35 minutes which shifted considerably (~ 3 hours) in 2OH-BNPP1+EGF treatment (Figure 5D).

There was no effect of 2OH-BNPP1 on Her2 degradation under these treatment conditions and time points in A549 and NCI-H358 cells (Figure 5A, 5C), further corroborating that BUB1 effect may be specific for EGFR and not for other EGFR family members.

### BUB1 inhibition reduces pEGFR endocytosis into EEA1 positive vesicles

**Figure 6:**
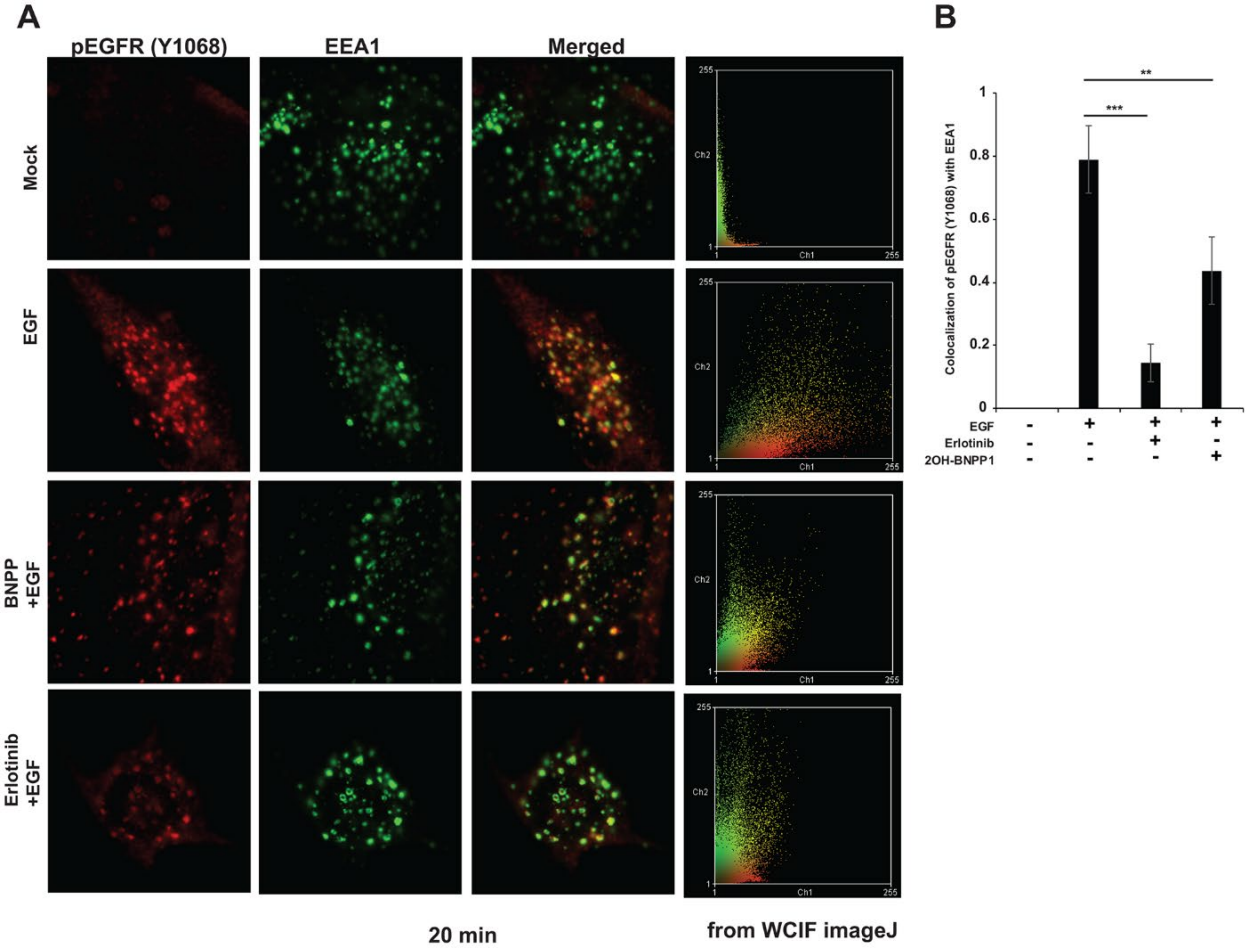
**BUB1 inhibitor reduces endocytosis of active EGFR in MDA-MB-231-1833 cells.** (**A**) Cells were plated on glass coverslips, serum starved for 3-4 hours and pretreated with 2OH-BNPP1 (10 μM) or erlotinib (10 μM) for 1 hour followed by EGF treatment (50 ng/mL). Cells were fixed at different time points (5, 20, 40 and 80 minutes) post EGF treatment and processed for staining with pEGFR (Y1068) and EEA1 antibodies. Representative confocal images 20 minutes post EGF treatment are shown. (**B**) co-localization of pEGFR (Y1068) with EEA1 was estimated on ImageJ using JACOMP plugin. Data at 20 min post EGF treatment is plotted. Two-sided students t-test was performed on MS-Excel (p values, ** = 0.00097, ***=3.17 X 10^-5^).

Since we observed that inhibition of BUB1 kinase activity by 2OH-BNPP1 reduced EGFR signaling, dimerization of active EGFR and enhanced EGFR half-life, we hypothesized that BUB1 may mediate these effects by altering the endocytosis of activated EGFR. MDA-MB-231-1833 cells were utilized to test this hypothesis. These cells were serum starved for 3 hours, then treated with vehicle (mock) or with 2OH-BNPP1 (10 μM) or with erlotinib (10 μM), followed by EGF stimulation (30-50 ng/mL). Cells were fixed and stained using pEGFR (Y1068) antibody. EEA1 antibody was used to evaluate endocytosis of EGFR in early endosomes. Confocal microscopy was performed after turning the beams on sequentially to avoid activation of fluorophores in another channel. Data was acquired for multiple planes and representative images with highest endosomes (EEA1 staining) is shown (Figure 6A). As expected, there was no measurable EGFR phosphorylation and endocytosis in starved cells (Figure 6A, top panel). Upon stimulation with EGF, EGFR was rapidly phosphorylated and colocalized with EEA1. Quantitation of the data indicated that about 80% of pEGFR colocalized with EEA1 in EGF stimulated samples (Figure 6B). Pre-treatment of cells with erlotinib or 2OH-BNPP1 reduced pEGFR endocytosis to about 20% or 40% respectively (Figure 6A, bottom panels, 6B). Protein thermal stability assay (melting curve analysis) confirmed that there was no direct target engagement between 2OH-BNPP1 and wild type-EGFR (Supplementary Figure 4). Osimertinib, which was used as a positive control protected EGFR at melting temperature (47° C; Supplementary Figure 4).

## DISCUSSION

The identification of BUB1 in an siRNA screen using the TGFBR1 kinase assay [24–27] was surprising since he majority of literature described BUB1 as the key component of the mitotic machinery where it ensures that chromatids are segregated with high fidelity. In support of our discovery on the potential role of BUB1 beyond chromatid segregation, recent studies have identified BUB1 as a component of signaling micro-domains within membranes [31–33]. Smith et al., [32] demonstrated that residues 4-313 of BUB1 interacted with residues 834-1291 of supervillin. Supervillin is a tightly bound membrane protein that provides a scaffold for interaction of signaling molecules. Specifically, supervillin co-fractionates with lipid rafts containing integrins and Rho GTPase. Chia et al., [33] identified BUB1 as a regulator of endosomal clathrin coated vesicles. Vollert et al., [34] and Cayrol et al., [35] confirmed this finding wherein a two-hybrid screen identified BUB1’s interaction with Vps5 and β2-adaptin, proteins involved in retrograde cycling of endocytic vesicles. Recently, a study identified a novel and important function of BUB1 at the plasma membrane to facilitate the virus to enter host cells through endocytosis [31]. This study identified that *Drosophila melanogaster* deficient in BUB1 became resistant to Drosophila C virus (DCV) infection which increased survival rates and reduced viral loads, compared to the wild-type control. Moreover, they found that BUB1 also functioned in the cytoplasm where it regulated clathrin-dependent endocytosis of DCV (and other pathogens). They found that DCV infection increased the interaction of BUB1 with clathrin adaptor on the cell membrane [31]. Similarly, BUB1 paralog BUBR1 was demonstrated to interact directly with insulin receptor (IR) and regulate IR endocytosis [36, 54]. These studies directly linked an important chromosome segregation complex to a key metabolic pathway which regulates homeostasis [36, 54]. All of these studies support our earlier findings on the moonlighting function of BUB1 in regulating TGF-β signaling at the cell membrane [27] and provided a strong foundation for the present studies.

Several studies have proved that like EGFR homodimers, ErbB2 homodimers are capable of endocytosis. It has been shown that EGFR can form both homodimers and heterodimers with ErbB2 following EGF stimulation. However, the EGFR–ErbB2 heterodimer was impaired in endocytosis [55]. These data indicate that receptor homodimerization rather than heterodimerization might improve EGFR internalization. That is why we limited our studies to study EGFR homodimers only. Recently Freed and colleagues deciphered a 2.9 Å crystal structure of EREG in complex with an EGFR extracellular dimer [56] which revealed a different structure from the previously reported TGFα/EGFR or EGF/EGFR dimers, suggesting that different EGFR ligands stabilize distinct EGFR conformations and lead to unique signaling. Therefore, we postulate that BUB1 may similarly regulate EGF driven EGFR signaling.

All the cell surface receptor tyrosine kinases (RTKs) undergo constitutive internalization and constitutive recycling from endosomes back to the cell surface. The rates of the endocytosis, recycling, and degradation regulate the half-life of an RTK protein [57]. Since we observed a marked increase in EGFR protein level upon siRNA mediated BUB1 depletion, we hypothesize that BUB1 protein (possibly as a scaffold) may regulate EGFR signaling through regulating the endocytosis, recycling or degradation of EGFR.

Based on thermal stability assay that 2OH-BNPP1 does not directly bind to recombinant EGFR, we postulate that the effect of this inhibitor on EGFR are due to the inhibition of BUB1 kinase activity. However, 2OH-BNPP1 has been recently shown to inhibit other kinases including PDGFRβ, CSF1R, VEGFR2, VEGFR3 [58], therefore we cannot rule out a possibility that the effect of 2OH-BNPP1 on EGF mediated EGFR signaling are not through these receptors. The endocytosis of PDGFRβ does not depend on its kinase activity [59] therefore we think that the 2OH-BNPP1 mediated effects on EGFR endocytosis are due mainly to the BUB1 kinase activity.

**Figure 7:**
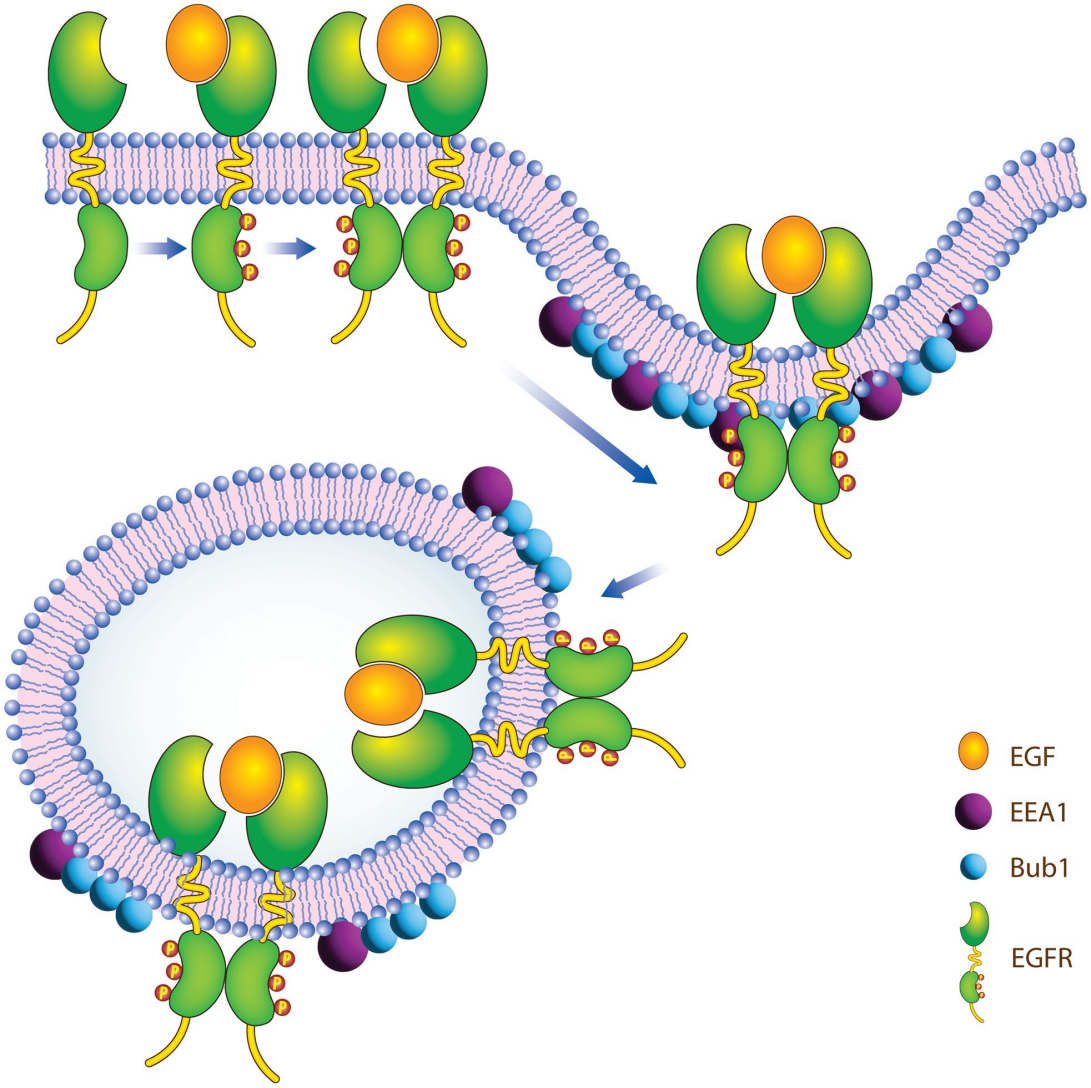
Proposed model of how BUB1 may regulate EGFR receptor endocytosis.

BUB1 has multiple domains in its N-terminal which play a role in its interaction with other proteins. These domains include tetratricopeptide repeats (TPR) domains (amino acid 99-132; [60]), Gli2-binding sequence (GLEBS)/BUB3 binding motif (residues 209-270; [61, 62]). BUB1 also has two KEN-boxes and a D-Box through which it interacts with CDH1 and CDC20 [63, 64]. It is possible that BUB1 uses one of these domains/motifs to interact directly or indirectly with EGFR and regulate EGFR endocytosis. It will be interesting to see if EGFR interacts with BUB1 directly or indirectly through proteins involved in endocytosis such as β-adaptin, Mig6, Sprt2 etc. Both, EGFR [65, 66] and BUB1 [34, 35] are known to associate with coated pit proteins adaptin, therefore we speculate that this could be one indirect way for these proteins to associate and for BUB1 to regulate EGFR endocytosis and signaling (Figure 7). Non-canonical Ser/Thr phosphorylation of EGFR has been shown to play a vital role in endocytosis of ligand nonbound EGFR monomer [67]. Since we observed an overall increase in total EGFR in BUB1 siRNA transfected cells (Figure 1, non EGF treated lanes) and since BUB1 is a Ser/Thr kinase, it is plausible that BUB1 phosphorylates EGFR and thus regulates EGFR endocytosis. We hypothesize that this could be an alternate mechanism through which BUB1 regulate EGFR signaling. All the cell lines used in the current study express wt-EGFR, it would be interesting to see how BUB1 protein or kinase inhibition affects EGFR signaling in cells that express mutant-EGFR. One of the limitations of this study is that we have not evaluated the effect of BUB1 ablation on EGFR signaling in mouse tumor models. It would also be interesting to access the effect of BUB1 inhibition with EGFR inhibition *in vivo*.

As summarized above several outstanding hypotheses will be tested in future studies (a) whether BUB1 directly interacts with EGFR if so through which domains, (b) whether BUB1 interacts indirectly to EGFR through β-adaptins, (c) whether BUB1 kinase activity or presence of BUB1 protein changes EGFR interaction with β-adaptins, Mig6 etc. thus impact endocytosis, (d) how BUB1 affects EGFR dimerization (switch between asymmetric and symmetric EGFR dimers), (e) whether BUB1 also affects EGFR-Her2 heterodimers, (f) how BUB1 inhibition increases EGFR degradation/turnover rate (t_1/2_), (g) whether BUB1 activity affects mutant-EGFR stability and downstream signaling, and (h) whether BUB1 inhibition improves chemotherapeutic potential of anti-EGFR agents in combination therapy regimes.

## CONCLUSIONS

We identified that BUB1 ablation reduces EGF mediated EGFR asymmetric dimers without affecting EGFR symmetric dimers leading to lowered EGFR endocytosis, EGFR degradation and downstream signaling in different cell-lines. The observed BUB1 mediated EGFR internalization and activation is EGFR-specific within the RTK family. We postulate that these observations may provide novel opportunities for therapeutic interventions for EGFR driven cancers.

## Supplementary Material

Supplementary Figures
