# Supplementary material for: Budding uninhibited by benzimidazoles-1 (BUB1) regulates EGFR signaling by reducing EGFR internalization
Source: Aging (Albany NY). 2023 Jul 3;15(13):6011–30. doi: 10.18632/aging.204820 (PMC10373970; doi:10.18632/aging.204820)
Supplement: Supplementary Figures [file aging-15-204820-s001.pdf]

SUPPLEMENTARY FIGURES

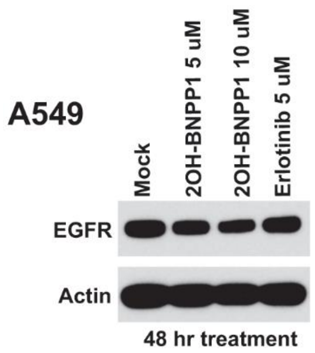

Supplementary Figure 1. BUB1 inhibition alone does not alter EGFR protein level. A549 cells were treated with 5 μM or 10 μM 2OH-BNPP1 for 48 hours. Erlotinib (5 μM) was used as a control in parallel lanes. Resulting lysates were probed with EGFR and Actin antibodies.

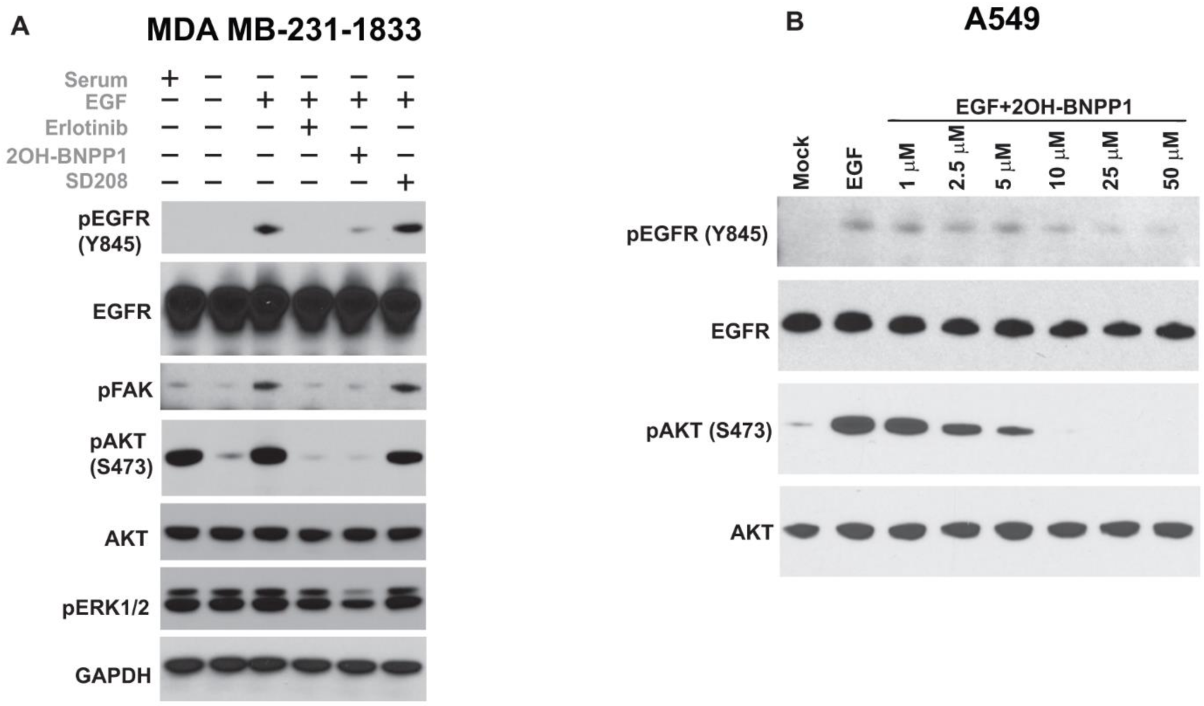

Supplementary Figure 2. (**A**) TGFBR1 inhibitor does not block EGFR signaling. MDA-MB-231-1833 cells were starved and pretreated with 10 μM 2OH-BNPP1 (BUB1 inhibitor), erlotinib (EGFR inhibitor) or SD208 (TGFBR1 inhibitor) for one hour followed by EGF (50 ng/mL) for an additional 30 minutes. Total cell lysates were resolved and probed with pEGFR (Y845), pFAK, pAKT (S473), pERK1/2 as well as total antibodies. (**B**) 2OH-BNPP1 dose-dependently inhibits EGFR signaling. A549 cells were starved and pretreated with different concentrations of 2OH-BNPP1 (1 μM to 50 μM) for one hour followed by EGF treatment (for 30 minutes). Resulting lysates were run on SDS-PAGE gels and probed using pEGFR (Y845), pAKT (S473) as well as antibodies against total proteins.

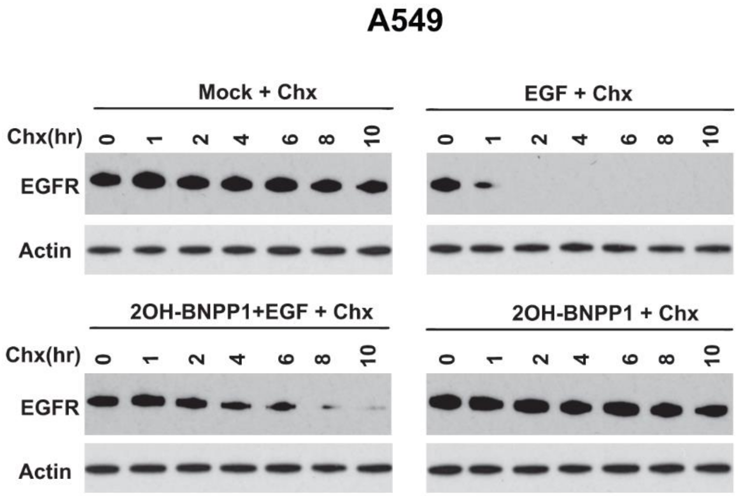

Supplementary Figure 3. 2OH-BNPP1 increase EGFR half-life. A549 cells were starved and were treated with vehicle (DMSO), or 2OH-BNPP1 (10 µM) and Cycloheximide (50 µg/ML) for 1 hour followed by EGF (50 ng/mL). Cells were harvested at different time points after EGF treatment (1-10 hours). Whole cell lysates made in denaturing lysis buffer were resolved on Bis-Tris SDS-PAGE gels in MOPS buffer. Proteins were transferred to PVDF membranes using Tris-Glycine transfer buffer containing 20% Methanol. PVDF were blocked with 5% milk-TBST and probed with total-EGFR and Actin antibodies.

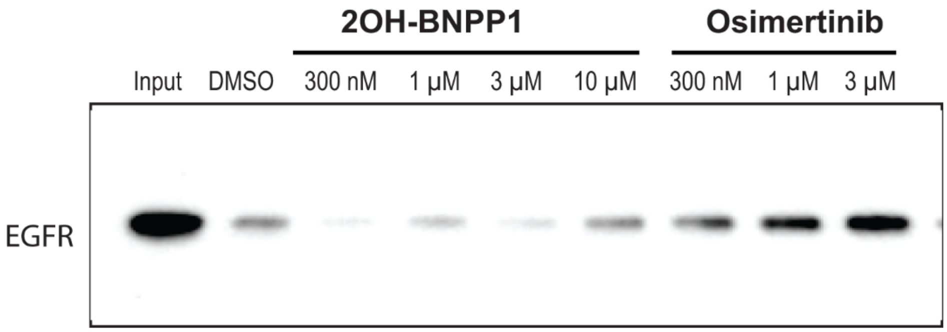

Supplementary Figure 4. 2OH-BNPP1 does not alter thermal stability of wt-EGFR. Baculovirally transduced recombinant wt-EGFR kinase domain (100 ng) was incubated with various concentrations of 2OH-BNPP1 (300 nM-10 µM) or vehicle (DMSO) in thin-walled tubes. Osimertinib (300 nM-3 µM) was used as a positive control in this experiment. These tubes were incubated at 4° C for 30 minutes followed by 3 minutes incubation at 47° C. The samples were centrifuged, non-precipitated parts (supernatants) were collected, denatured with 2X Laemmli buffer and resolved on SDS-PAGE gels. This melting curve analysis detected no shifts in the melting curve of EGFR with 2OH-BNPP1 indicating that 2OH-BNPP does not directly bind to EGFR.
